# Efficacy and Optimal Dose of Botulinum Toxin A in Post-Stroke Lower Extremity Spasticity: A Systematic Review and Meta-Analysis

**DOI:** 10.3390/toxins13060428

**Published:** 2021-06-18

**Authors:** Thanh-Nhan Doan, Mei-Ying Kuo, Li-Wei Chou

**Affiliations:** 1 Department of Physical Therapy and Graduate Institute of Rehabilitation Science, China Medical University, Taichung 406040, Taiwan; drchannhan@gmail.com (T.-N.D.); mykuo@mail.cmu.edu.tw (M.-Y.K.); 2 Department of Rehabilitation, Quang Nam Northern Mountainous Region General Hospital, Quang Nam 560000, Vietnam; 3 Department of Physical Medicine and Rehabilitation, China Medical University Hospital, Taichung 404332, Taiwan; 4 Department of Physical Medicine and Rehabilitation, Asia University Hospital, Asia University, Taichung 413505, Taiwan

**Keywords:** Botulinum toxin, stroke, spasticity, lower extremity, optimal dose

## Abstract

Post-stroke spasticity impedes patients’ rehabilitation progress. Contradictory evidence has been reported in using Botulinum Neurotoxin type A (BoNT-A) to manage post-stroke lower extremity spasticity (PLES); furthermore, an optimum dose of BoNT-A for PLES has not yet been established. Therefore, we conducted a systematic review and meta-analysis of randomized controlled trials (RCTs) to identify the efficacy and optimal dose of BoNT-A on PLES. "Meta" and "Metafor" packages in R were used to analyze the data. Hedges’ g statistic and random effect model were used to calculate and pool effect sizes. Twelve RCTs met the eligibility criteria. Muscle tone significantly improved in week four, week eight, and maintained to week twelve after BoNT-A injection. Improvements in functional outcomes were found, some inconsistencies among included studies were noticed. Dosage analysis from eight studies using Botox® and three studies using Dysport® indicated that the optimum dose for the commonest pattern of PLES (spastic plantar flexors) is medium-dose (approximately 300U Botox® or 1000 U Dysport®). BoNT-A should be regarded as part of a rehabilitation program for PLES. Furthermore, an optimal rehabilitation program combined with BoNT-A management needs to be established. Further studies should also focus on functional improvement by BoNT-A management in the early stage of stroke.

## 1. Introduction

Post-stroke lower extremity spasticity (PLES) has been being a challenging issue in the rehabilitation field, with the prevalence ranging from 17% to 42.6% [1]. Albeit a few positive influences of spasticity have been reported, it is irrefutable that this upper motor neuron syndrome’s component has clinically negative influences on health-related quality of life [2]. PLES was consistently demonstrated to be negatively correlated with ambulation, functional capacity, and balance ability [3,4,5,6]. Inappropriate distribution of pressure during weight-bearing is attributable to foot pain, callus formation, and soft tissue breakdown [7]. While hip flexors and knee extensors strength predominantly affect gait speed, the degree of ankle plantar flexors spasticity primarily influenced the temporospatial gait performance of hemiplegic patients [8]. Botulinum Neurotoxin type A (BoNT-A) produced by gram-positive bacteria named Clostridium botulinum is the most popular and well-established therapeutic application globally with three leading products: onabotulinumtoxinA (Botox®), abobotulinumtoxinA (Dysport®), and incobotulinumtoxinA (Xeomin®) [9]. Although differing in nontoxic accessory proteins, these preparations have identical neurotoxin structure and similar mechanisms of action, which works by inhibiting the release of acetylcholine in neuromuscular junctions [10]. Despite the lack of consensus in terms of dosage conversion ratio among products [11], the available data on practical application suggests an approximate conversion ratio Dysport®: Botox® (or Xeomin®) of 3:1 could be implemented for several movement disorders, including spasticity [10,12]. Several randomized controlled trials (RCTs) have been performed to investigate the effect of BoNT-A for PLES with conflicting results either on muscle tone or other functional outcomes [13,14,15,16]. Foley et al., 2010 performed a meta-analysis, concluding that BoNT-A significantly improved gait velocity in PLES patients [17], whereas the other meta-analysis concluded that BoNT-A had no significant efficacy on lower extremity muscle tone and gait speed [18]. Furthermore, an optimum dose of BoNT-A for PLES has not yet been established. Therefore, we conducted this systematic review and meta-analysis of randomized controlled trials to identify the efficacy and optimal dose of BoNT-A on post-stroke lower extremity spasticity. 

## 2. Results

### 2.1. Study Selection

**Figure 1:**
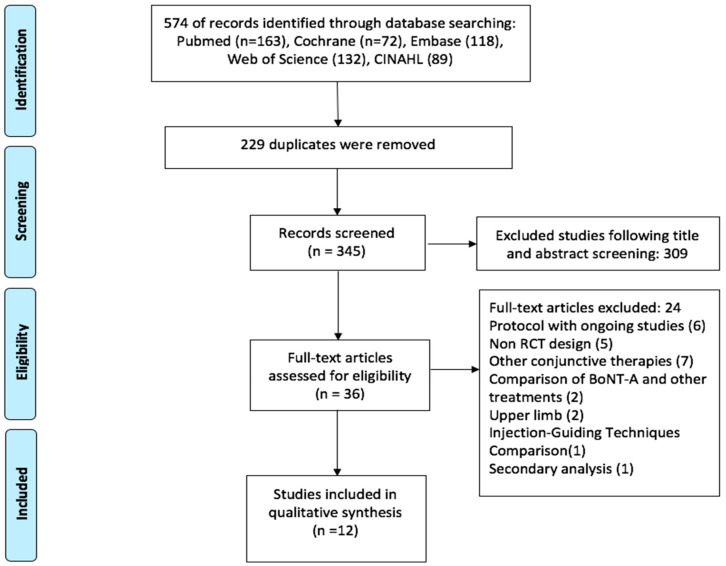
Study flow diagram.

We presented a PRISMA flow chart of the selection process in Figure 1. The original search for this systematic review was run from inception up to February 2021. Initially, 574 articles were found from five electronic libraries, then 229 duplicates were excluded. After reviewing titles and abstracts, 309 apparent irrelevant papers were removed. Of these 36 remainders, we then excluded 24 studies for several reasons: protocol with ongoing studies (n = 6), non-RCT design (n = 5), other conjunctive therapies combined with BoNT-A injection (n = 7), comparison of BoNT-A and other treatments (2), upper limb (n = 2), injection-guiding techniques comparison (n = 1), secondary analysis (n = 1). Eventually, 12 studies fulfilled the eligibility, in which six studies were randomized placebo-controlled trials [13,15,19,20,21,22], three studies were randomized, placebo-controlled, dose-ranging studies [14,16,23], and three studies were randomized dose-ranging study [24,25,26]. All included studies assessed by the PEDro scale gained a score range of 9 or 10 of good quality, the risk of bias assessment was detailed in Table S1. Of the 12 included studies, two were conducted in France [13,22], two in China [20,26], three were multi-centre, multinational trials [16,21,23]. The remaining five studies were conducted in Germany [19], Japan [15], Italy [24], Australia [14], and Brazil [25]. The majority of trials used BoNT-A for PLES in the chronic stage; only 75 patients in two studies received BoNT-A injection starting less than three months after the event [19,20]. The characteristics of the 12 included studies are detailed in Table 1.

### 2.2. Qualitative Analysis

#### 2.2.1. Efficacy of BoNT-A in Post-Stroke Lower Extremity Spasticity

Most studies included have used Ashworth Scale or Modified Ashworth Scale (MAS) to assess spasticity; only one trial aside from MAS, calculated surface electromyography (sEMG) levels during passive movement of the ankle for the examination of the gastrocnemius spasticity [20]. Muscle tone was analyzed at various time points after the injection of BoNT-A. At 4-week assessments, significant improvements in MAS scores of calf muscles were found in two Phase III trials [21,23] and three other studies [13,15,22]. MAS scores of three other studies showed substantial spasticity improvements compared to placebo eight weeks after injections [15,16,20]. The trial analyzed the gastrocnemius’ sEMG level, indicating significant differences in muscle tone improvement between treatment and control groups at week four and week eight evaluations [20]. BoNT-A also produced significant ankle spasticity reduction compared to placebo at week twelve in two studies [16,19], whereas conflicting results were found in the other studies [14,15]. 

#### 2.2.2. Efficacy of BoNT-A in Functional Outcomes

Regarding motor function, Fugl-Meyer scores at the twelfth week [13] and the eighth week [20] after intervention were significantly higher in the treatment group than in the placebo group. In contrast, the leg and trunk section of Rivermead Motor Assessment was evaluated with an insignificant improvement compared to placebo at all time point assessments after BoNT-A administration [16]. In terms of functional ambulation, five studies have found no significant improvement in gait velocity following BoNT-A injection compared to placebo injection [13,15,16,22,23]. Functional Ambulation Category was used to compare functional mobility following BoNT-A versus placebo injections, which indicated no significant difference between the two groups [22]. Meanwhile, the Physicians Rating Scale based on video recordings of the patients’ gait was used to qualitatively rate gait quality, suggesting that patients’ gait quality after intervention in the BoNT-A group was better than in the placebo group [14]. Furthermore, significant improvements in gait parameters such as step length, cadence, as well as gait speed were observed at eight weeks following the early intervention of PLES with BoNT-A [20].

Active ankle dorsiflexion significantly improved four weeks [13] and twelve weeks [14] after the intervention in the BoNT-A group compared to the placebo group. Meanwhile, the study of Pittock et al., 2003 showed no statistically significant differences between the interventional and control groups for this aspect of ankle movement [16].

Patients’ satisfaction reported objectively by themselves were noticed a considerable difference in favor of the BoNT-A group than the control group [13]. Wein et al., 2018 employed the Goal Attainment Scale to follow the improvement in patients’ individual goals, reporting significant improvement in the treatment group than in the placebo group. Clinician Global Impression (CGI) assessed by physicians was significantly better in the treatment group compared to the placebo group; moreover, CGI was strongly correlated with the improvements in ankle spasticity (MAS scores) at all double-blind time points [21].

Regarding balancing ability, Kerzoncuf et al [22] conducted a study with the primary outcome as the assessment of postural sway using an AMTI force plate. BoNT-A group significantly decreased body sway compared to the placebo group; moreover, during the interval of two months following injections, the occurrence of falls in the BoNT-A group was lower than those in the placebo group [22].

In regard to activities of daily living (ADLs), early BoNT-A intervention for PLES produced considerable improvements in Modified Barthel Index (MBI) [20], whereas BoNT-A injections for chronic PLES did not improve the Functional Independence Measure score [22].

#### 2.2.3. Dosage and Target Muscles

There are two main preparations used in the majority of included studies: OnabotulinumtoxinA (Botox®) and AbobotulinumtoxinA (Dysport®). The doses ranged from 100 U to 540 U of Botox®, while 500U, 1000 U, and 1500 U were the three major doses of Dysport® used in the included studies. Despite having some minor inconsistencies in choosing target muscles, the main treated muscles in the included studies were gastrocnemius, soleus, and tibialis posterior. The details of the total dose and the amount of BoNT-A injected in each muscle among individual trials are presented in Table 2 and Table 3.

Mancini et al., 2005 compared three mean doses of Botox®: 167 U, 322 U, 540 U, and placebo in 234 chronic stroke participants, the medium dose (322 U) was found to be effective and safe for PLES, the high-dosage group showed the highest occurrence of adverse effects during the four weeks after treatment [24].

Dunne et al., 2012 and Pimentel et al., 2014 carried out comparative studies between different doses of Botox®; 300 U was compared to 200 U and 100 U, respectively. While Pimentel et al., 2014 concluded that 300 U Botox® produced a significantly greater reduction in muscle tone than 100 U regimen at two, four, eight, and twelve weeks after injection. Dunne et al., 2012 indicated that there was no significant difference between 200 U and 300 U regimens in alleviating spasticity at the twelfth-week assessment; however, when analyzing participants with more severe spasticity at baseline defined by Ashworth Scale Scores ≥3, a statistically significant difference was found, favoring the regimen of 300 U [14,25]. The therapeutic effect of 300 U Botox® in improving lower extremity spasticity was confirmed in two other studies [15,21]. In the other multicenter randomized, double-blind study conducted in France, 40 chronic stroke subjects were randomized to receive either placebo or a maximum of 300 U Botox® (mean 227 U) flexibly based on the physician’s adjustment for each patient, the results of this study showed that patients who received Botox significantly improved not only in spasticity but in balancing ability [22].

Two other trials were performed to evaluate Botox®’s efficacy in attenuating calf muscle spasticity in the early stage after stroke, in which fixed-dose regimens injected into mandatory muscles for every subject were applied [19,20]. Fietzek et al., 2014 used the total dose of 230 U Botox® for spastic equinovarus within the first three months into medial gastrocnemius head (60 U), lateral gastrocnemius head (30 U), soleus (70 U), and tibial posterior (70 U). The target muscles based on anatomical landmarks without any supplementary guidance technique were applied and kept similar in all patients. The assessments of muscle tone at week four showed no significant difference between the two groups. It was not until the twelfth week that subjects who received BoNT-A injection significantly improved their MAS score [19]. Tao and colleagues, 2015 conducted the trial in which twenty-three patients who had suffered from stroke within the first six weeks were randomized to receive either placebo or each 50 U Botox® into four muscles (the medial head of gastrocnemius, the lateral head of gastrocnemius, soleus and tibialis posterior), making up a total dose of 200 U. The injection was guided by electrical stimulation technique. A comprehensive rehabilitation program was performed in both groups following the intervention. Outcomes of muscle tone assessed either by MAS score at week eight or sEMG at week four and week eight of patients in the treatment group significantly improved compared to those in the control group. More importantly, significant improvements were also found in gait speed, step length, and cadence compared to the control group in the eighth week. Motor function and quality of life also considerably improved through the assessments of FMS and MBI in contrast to the placebo group at eight weeks following injections [20].

Three dosage levels of Dysport®: 500 U, 1000 U, 1500 U were selected to treat lower limb spasticity in three studies (Table 3). Pittock et al., 2003 performed a study comparing the therapeutic effects of these three doses compared with placebo; in this study, 62.5% and 37.5% of each group’s total dose were injected into gastrocnemius and soleus, respectively. The results of this study proved that the dose of 500 U is not enough to produce therapeutic efficacy. Compared to placebo, the most significant alleviation in spasticity was seen in the group receiving Dysport® at 1500 units, but it should be cautious because they also produced excessive muscle weakness in some individuals. Even though the results in the reduction of spasticity level were not as good as the 1500 U group, the dose of 1000 U also had significant efficacy in attenuating spasticity [16].

Burbaud., 1996 administered a dose of 1000 U Dysport® primarily into three muscles gastrocnemius, soleus, and tibialis posterior, of which more than half of the total dose (500 to 1000 U) was injected into the gastrocnemius muscle, this study showed a significant improvement in muscle tone (MAS scale) compared to the control group [13]. Gracies et al., 2017 undertook a dose-ranging placebo-controlled study comparing 1000 U and 1500 U of Dysport® efficacy. In this study, remarkably, only one-fifth of the total dose was injected into gastrocnemius, one-third was injected into soleus, the remainder was treated to optional muscles selected by the investigator. This study suggested that only the group that received 1500 U had a significant reduction in MAS of gastrocnemius–soleus complex compared to the placebo, whereas the decrease in soleus’s MAS score was significant in both the 1000 U and 1500 U groups compared to the control group. Additionally, results after repeated injections in one-year open-label were similar across both doses. Regarding safety in this study, it is also worth noting that while muscular weakness events in the placebo and the 1000 U groups were local, there were three episodes of regional weakness and three cases of generalized muscle weakness in the 1500 U group [23].

A study conducted in China in 2017 took into consideration not only the dosage but also the diluted concentration of their own BoNT-A product named HengLi® (Lanzhou Institute of Biological Products, Lanzhou, China). One hundred and four PLES patients were randomized into four groups based on two doses (200 U and 400 U) and two concentrations (50 U/mL and 100 U/mL). MAS score, 10-meter timed walking test, the 6-meter timed up and go were assessed at four days, one week, two weeks, four weeks, and twelve weeks following the injections. The onset time in spastic improvement and the duration of therapeutic effect up to 6 months were also recorded in individuals in each group. The most rapid therapeutic onset (one week after treatment) belonged to two high-dose groups in which, 79% of patients in the high-dose/low-concentration group, compared to 64% of the high-dose/high-concentration group, improved their muscle tone within 3–10 days after the treatment. At two weeks and four weeks, MAS scores were significantly lower than baseline in all four groups. Results of the 10-meter timed walking test and 6-meter timed up and go in comparison with those before and at two weeks after treatment significantly improved in both the high-dose groups. In the twelfth week, only MAS scores in both high-dose groups remained significantly improved. 79% of patients in the high-dose/low-concentration group maintained efficacy until five months compared with 86% in the high-dose/high-concentration group, while 17% of patients in the high-dose/low-concentration continued to prolong the therapeutic effect beyond six months compared with 5% of those who were in high-dose/high-concentration group [26]. 

#### 2.2.4. Safety

Two studies using the early intervention of Botox® with the dose of 200 U and 230 U reported that during the study period, adverse effects did not happen [20] and no adverse effects were treatment-related [19], respectively. Other studies that used 300 U of Botox® reported that there were insignificant differences in terms of adverse effects’ incidence between the treatment and the placebo groups [14,15,21,22]. The mean total dose of 540 U Botox® produced more severe and prolonged adverse effects, leading to significant reductions in gait velocity and muscle strength compared to the two lower dose groups in the assessment at week four post-intervention. At month four after the intervention, the injected muscle strength remained significantly lower in the highest dose group compared to the assessment at the baseline [24]. One thousand units of Dysport® were used to treat plantar flexor spasticity subsequently reported that apart from injection side pain, no generalized or localized adverse effects had occurred [13]. Although no statistical between-group comparison was conducted with respect to adverse effects in two randomized placebo-controlled, dose-ranging studies, these two studies reported that 1500 U of Dysport® for lower extremity spasticity had produced excessive muscle weakness or remotely spread of toxin in some individuals [16,23].

### 2.3. Quantitative Analysis

#### 2.3.1. Efficacy of BoNT-A in Lower Extremity Spasticity

Meta-analyses of muscle tone assessments at week four, week eight, and week 12 post-injection were conducted. At week four and week 12, the included studies assessed muscle tone by Ashworth Score or Modified Ashworth Score, which was therefore deemed appropriate to calculate the SMD. At week eight, two trials were containing muscle tone assessment data using the same tools; hence, we computed the Mean Difference (MD) at this time point. 

**Figure 2:**
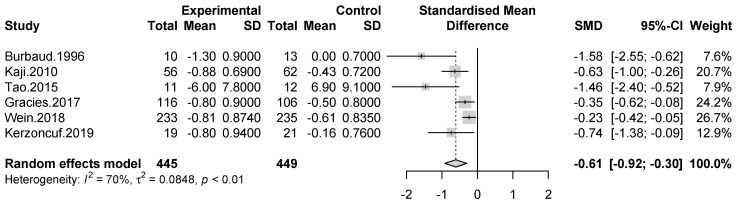
Meta-analysis of muscle tone assessments at week four after treatment.

Data from spasticity assessment at week four were available in six studies. A significant effect size was observed in the improvement of muscle tone in the interventional group compared to control group (SMD = −0.61 [95% confidence interval: −0.92; −0.3]; *p* < 0.0001, I^2^ = 70%). (Figure 2)

**Figure 3:**
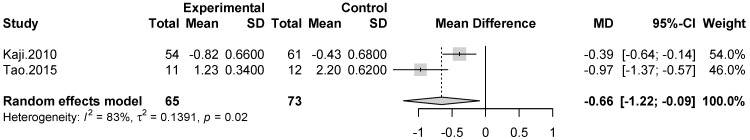
Meta-analysis of muscle tone assessments at week eight after treatment.

At eighth week after the intervention, muscle tone was significantly improved in BoNT-A groups when compared to control group (MD = −0.66 [95% confidence interval = −1.22; −0.09]; *p* = 0.02, I^2^ = 83%). (Figure 3)

**Figure 4:**
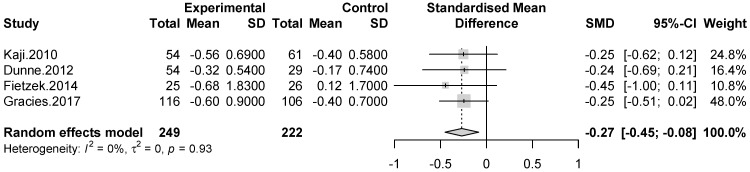
Meta-analysis of muscle tone assessments at week 12 after treatment.

Meta-analysis including four trials demonstrated a significant efficacy without heterogeneity in favor of BoNT-A compared to placebo remained to week 12 after the intervention (SMD = −0.27 [95% confidence interval = −0.45; −0.08]; *p* = 0.0041, I^2^ = 0%). (Figure 4).

#### 2.3.2. Efficacy of BoNT-A on Functional Outcomes

**Figure 5:**
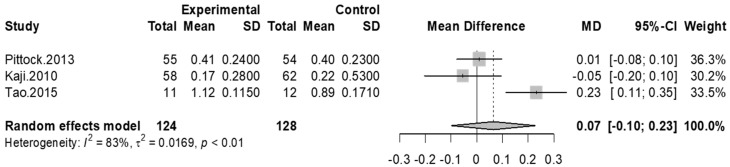
Meta-analysis of gait velocity at week 8 after treatment.

Four studies assessed gait velocity pre and post-injection of BoNT-A and placebo groups, three of which had available data to convert gait speed to meters per second (m/s). At fourth-week and twelfth-week assessments, all studies indicated that there was no significant difference between control and BoNT-A injection groups for improving gait speed, accordingly, we did not conduct meta-analyses at these time points. The result of the meta-analysis at the eighth-week assessment showed a small improvement in gait velocity but not statistically significant in the experimental group when compared to the placebo group (MD = 0.07 [95% confidence interval: −0.1; 0.23]; *p* = 0.43, I^2^ = 83%). (Figure 5).

Most of the included studies focused on the improvement of spasticity and few trials concerning the motor function, ADLs, or balancing ability; therefore, we did not conduct meta-analyses for these outcomes, data were synthesized narratively.

#### 2.3.3. Publication Bias Analysis

Publication bias is noticed with the result of Egger’s test for funnel plot asymmetry (*p* = 0.0002), which is statistically significant in the assessments of muscle tone of included studies at all time points. The funnel plot was manifested in Figure S1.

## 3. Discussion

### 3.1. Main Findings 

This systematic review and meta-analysis are to clarify the efficacy and the optimal dose of Botulinum toxin A on post-stroke lower extremity spasticity. Basing on the evidence from the included studies, we found that:

1. Main Finding 1: Botulinum Toxin A Effectively Improves Post-Stroke Lower Extremity Spasticity.
2. Main finding 2: The doses of approximately 300U of Botox® or 1000 U of Dysport® are the most preferable for the commonest pattern of post-stroke lower extremity spasticity, which is spastic plantar flexors.

### 3.2. Efficacy of BoNT-A in Improving Spasticity

This systematic review and meta-analysis analyzed 12 RCTs that utilized BoNT-A injection in PLES. The meta-analyses’ results have proven that the management with BoNT-A significantly surpassed placebo in attenuating muscle tone assessments four weeks, eight weeks, and three months after the intervention. It is reasonable to explain that the considerable heterogeneity in the assessments of the fourth and eighth weeks is caused by differences in duration of spasticity, dose distribution, and concomitant rehabilitation program among studies in which a massive improvement in spasticity was observed in the small studies applying BoNT-A in combination with a comprehensive rehabilitation program and early on. Indeed, when excluding two small studies [13,20] in subgroup analysis, the result of a meta-analysis of muscle tone assessment at week four remains significant with SMD: −0.36 [−0.58; −0.15], *p* = 0.0008; with heterogeneity significantly reduced (I^2^ < 50%, *p* = 0.13). The forest plot of the subgroup analysis was shown in Figure S2. Sun et al., the authors combined data of lower extremity muscle tone assessment from five studies in one meta-analysis regardless of different time points of assessment. One of their five included trials did not have a placebo control group. One other study included two groups, both the interventional and control groups were treated with BoNT-A. As a result, there was no significant effect of BoNT-A observed in their meta-analysis [18]. Contrary to their study, in our present study, we only collected RCTs, which employed BoNT-A in comparison with placebo to conduct meta-analyses of muscle tone assessment at the same time points of evaluation.

### 3.3. Efficacy of BoNT-A on Functional Outcomes

#### 3.3.1. Efficacy of BoNT-A on Functional Outcomes for Post-Stroke Lower Extremity Spasticity at the Chronic Stage

BoNT-A was proven effective in improving motor function assessed by Fugl-Meyer score [13,18], balancing ability, and reducing the occurrence of falls [22] as well as dependency on walking aids [16]. Although treatment with BoNT-A did not show significant improvement in gait velocity compared to placebo, an improvement was observed in gait quality as assessed by the Physicians Rating Scale using a qualitative rate based on video recordings of the patient’s gait [14]. The previous meta-analysis including eight studies has concluded that BoNT-A treatment for PLES significantly improved gait velocity. It is worth noting, however, that only two of the eight studies were randomized controlled trials with BoNT-A and placebo groups, data of pre- and post-treatment from six other studies were also pooled in a meta-analysis. This limitation, therefore, undoubtedly compromised the precision of the effect estimate that the authors reported [17]. Gait velocity is just one component of gait quality, and it might not be sensitive enough to capture improvements comprehensively. It should not be a single outcome measure for gait quality [17].

#### 3.3.2. Efficacy of Early BoNT-A Intervention for Post-Stroke Lower Extremity Spasticity 

It appears that the improvements in motor function, functional ambulation, and patient’s ADLs are achievable if an early injection of BoNT-A is administered in conjunction with a comprehensive rehabilitation program [20]. However, this suggestion should be confirmed by further studies with a larger sample size and longer follow-up duration.

Secondary analysis of a phase 3, randomized double-blind study, 468 patients were stratified by time, subjects who received BoNT-A injection ≤ 24 months after stroke had greater improvements in Modified Ashworth Scale and Goal Attainment Scale than those >24 months since stroke [27].

#### 3.3.3. The Main Muscles of the BoNT-A Injection for Post-Stroke Lower Extremity Spasticity and the Optimal Dose

Botox® dosage of approximately 300 units has been proven to be effective and safe compared to placebo and its other doses in PLES treatment in the chronic stage [14,15,21,22,24,25]. This finding is consistent with the consensus of ten expert clinicians in physical medicine and rehabilitation and neurology from a Delphi Panel Approach [28].

The commonest PLES pattern was the equinovarus or equinus; accordingly, the gastrocnemius-soleus complex (GSC) is the most frequently treated muscle group; in addition, patients with lower limb spasticity may experience concurrent spasticity in other muscle groups. Personalization in choosing the muscle group for treatment is, therefore, essential [29]. However, when the GSC was identified as the patient’s primary spasticity, this muscle group should be treated with a sufficient dose of BoNT-A. When considering three studies using Dysport® in treating PLES [13,16,23] (Table 3), researchers were initially interested in the GSC’s spasticity and chose it as an inclusion criterion. These three studies had 1000 U Dysport® treated groups, which were compared to placebo. While the first two studies utilized a more considerable quantity of Dysport®: 500–1000 U [13] or 625 U [16] for the gastrocnemius muscle, only 200U was injected into this muscle in the study of Gracies et al., 2017 [23]. The quantity of medication injected into the soleus was relatively similar across these three studies. Consequently, the first two studies showed a significant improvement in GSC spasticity, whereas such improvement was not observed in the third study. It is unreasonable to conclude that the 1000 U of Dysport® is insufficient to attenuate the spasticity of GSC.

#### 3.3.4. Dilution

Regarding dilution, one included study investigated the dilution effects of Hengli®- the product of BoNT-A, which was reported having comparable dose and efficacy with Botox®. This study concluded that the concentration of 2ml per 100U Hengli® is preferable for their domestic product in PLES treatment [26]. The dilution of 2ml per 100U of Botox® was used in three included studies [19,24,25] which is consistent with the Botox® manufacturer’s recommendation. Higher dilution of 4ml per 100U Botox® [21] or 5ml per 100U Botox® [14,15] was also used in the included studies. It is assumed that the greater dilution for larger muscles may produce better therapeutic effects than the low diluted volume, but there has been no evidence to prove it [28].

The dilution ratio of Dysport® in the three included studies were 200U/mL [13]; 125U/mL, 250U/mL, 375U/mL [16]; 133U/mL, 200U/mL [23]. According to the manufacturer’s instruction, the dilution could be ranged from 100U/ml to 500U/ml. We had no evidence in regards to the preferable dilution volume of Dysport® for post-stroke lower extremity spasticity.

#### 3.3.5. Injection-Guiding Techniques

The majority of the included studies used EMG, ES, or ultrasound as injected guidance. There were multifactorial influences on the results of these studies. Therefore, we do not make any comparisons about the effect of the instrumented techniques among them. However, there is reliable evidence suggesting that injection-guiding techniques enhance the efficacy of BoNT-A in treating spasticity compared to manual needle placement [30].

### 3.4. Limitation

Several limitations should be mentioned in the current study. Firstly, we aimed to investigate the efficacy of BoNT-A on lower extremity spasticity. However, most RCTs that have met the eligibility predominantly used BoNT-A to treat spastic equinus or equinovarus deformity. Secondly, we have recruited 12 studies in this systematic review, in which only nine studies had available data for meta-analyses; moreover, there were inconsistencies in time points of assessment of the outcomes after the intervention among included studies. Publication bias, therefore, might influence the results of meta-analyses. Thirdly, the available data of muscle tone assessment in the included studies were just up to twelve weeks after the intervention, therefore it is unknown the longer efficacy of BoNT-A treatment for PLES beyond that period of time. Finally, dosage conclusions were only based on qualitative analysis.

## 4. Conclusions

The findings of this systematic review and meta-analysis verify the efficacy of BoNT-A in improving lower extremity spasticity following stroke. The doses of approximately 300 U of Botox® and 1000 U of Dysport® appear to be the most favorable for spastic plantar flexors. Further studies are needed to confirm the functional improvement of BoNT-A management in the early stage. Evaluations should not only concentrate on gait velocity but also on gait quality or balancing ability. The optimal concomitant rehabilitation regimen needs to be established and presented in detail.

## 5. Methods

### 5.1. Eligibility Criteria

Studies were eligible if they were randomized controlled trials (RCTs) published in English, which recruited stroke patients with lower extremity spasticity. We accepted any products of BoNT-A as the intervention; however, we excluded studies assessing the efficacy of BoNT-A in combination with other modalities (such as orthotics, extracorporeal shock wave therapy, magnetic stimulation, therapeutic ultrasound, or electrical stimulation, and so forth). We accepted placebo or different doses of BoNT-A as the control group. Muscle tone assessments were used as the primary outcome, and secondary outcomes could be measures related to motor functions, functional ambulation, daily living activities, balance, and satisfaction with the treatment.

### 5.2. Search Strategy and Screening Process

A systematic search was conducted up to 10 February 2021 of Pubmed, EMBASE, Web of Science, CINAHL, and Cochrane libraries with two primary terminologies: “Botulinum toxin type A” and “Lower extremity spasticity”. The search terms are detailed in Appendix A. We also scanned cited lists of the relevant studies to retrieve potential RCTs, studies were not available in full-text or missing data were requested directly by contacting the corresponding authors via email. Initially, duplicates were automatically removed by EndNote X9, and the apparent irrelevant studies were excluded through reviewing titles and abstracts. Two authors independently read full texts of potential articles to obtain studies that met the eligibility. All hesitations were resolved by discussion with the third author.

### 5.3. Assessment of the Risk of Bias

The Physiotherapy Evidence Database (PEDro) scale was utilized to estimate the risk of individual studies bias with the total score ranging from 0 to 10. Two authors independently rated each trial. In cases of discrepancies of rating, the consensus was made by discussion. Trails scored ≥ nine is considered high quality, six to eight is considered good quality, studies that scored four to five are fair quality, and below four is reflected the low quality [31]. 

### 5.4. Data Extraction

We extracted, for each study, the study characteristics (author’s name, year of publication), participant characteristics (country, mean age, time since stroke), BoNT-A interventions (preparations, dosage, dilution, injection technique), concomitant rehabilitation program (percentage of patients receiving rehabilitation program, intensity, duration, frequency of rehabilitation program), outcome assessments (muscle tone and secondary outcome of interest) at the same time points of assessment. The first reviewer extracted data, then the second author checked for accuracy and completeness again.

### 5.5. Statistical Analysis

“Meta” and “Metafor” packages in the statistical software R version 3.6.2 (R Foundation for Statistical Computing, Vienna, Austria) were used to analyze the data. The effect sizes were calculated via means and standard deviations in each study according to Hedges’ g statistic. Regarding quantitative outcomes using the same measurement, we pooled mean difference (MD). For outcome assessment tools with various versions or different measures, we calculate the standardized mean difference (SMD) to measure the effect sizes of the included studies. The random-effect model was constructed to pool the effect sizes because of the assumption that clinical heterogeneity among the included studies was likely. The I^2^ statistic was used to analyze the heterogeneity among included studies, I^2^-values of 75%, 50%, and 25%, corresponding to high, moderate, and low levels of heterogeneity [32]. We used forest plots to display effect sizes, confidence intervals, pooled effect size, and heterogeneity. Publication bias was assessed using funnel plot and Egger’s test [33]. The statistical significance was defined as *p* < 0.05. 

## Figures and Tables

**Table 1:** Characteristics of included studies.

First Author, Year	Sample Size	Event Duration	Preparations and Doses	Guidance Techniques	Concomitant Rehabilitation	Control	Outcomes	Results
Burbaud. 1996 [13]	23	>3 months	Dysport®: 1000 U	EMG	All patients continued with active physiotherapy	placebo	Ashworth scale (AS) Fugl–Meyer score (FMS) Gait velocity (GV) Active dorsiflexion (AD)	AS + FMS + GV – AD+
Pittock. 2003 [16]	234	>3 months	Dysport®: 500 U, 1000 U, 1500 U	None	38% received physiotherapy, most only received 1 session	placebo/ dose-ranging	Modified Ashworth Scale (MAS) Use of Walking Aids (WA) Gait velocity (GV) Active dorsiflexion (AD) Rivermead Motor Assessment (RMS)	MAS + WA + GV – AD– RMS –
Mancini. 2005 [24]	45	12–36 months	Botox®: 160 U, 320 U, 540 U	EMG	Physical therapy was discontinued two months before the study and was suspended during the study period	dose-ranging	Modified Ashworth Scale Gait velocity Medical Research Council Scale	Medium dosage is safe and effective
Kaji. 2010 [15]	120	>6 months	Botox®: 300 U	EMG	Rehabilitation programs were not specified	placebo	Modified Ashworth Scale (MAS) Gait velocity (GV) Clinical global impression (CGI)	MAS + GV – CGI +
Dunne. 2012 [14]	83	>6 weeks	Botox®: 200 U, 300 U	EMG or ES	41% of participants receiving unspecified physiotherapy at the time of study enrollment, not mentioned during the study period	placebo/ dose-ranging	Ashworth Scale (AS) Gait quality (GQ) Leg spasms (LS) Active dorsiflexion (AD)	AS – GQ + LS + AD +
Pimentel. 2014 [25]	21	>6 months	Botox®: 100U, 300 U	None	All participants underwent a rehabilitation program included PT (aquatic physiotherapy, motor physiotherapy to improve gait and range of motion), OT with the goal of training ADL, these programs were trained at least 4 days/ week for at least 40 minutes/day.	dose-ranging	Modified Ashworth Scale (MAS)	Improved spasticity in the 300U group was better than in the 100U group.
Fietzek. 2014 [19]	52	<3 months	Botox®: 230 U	None	All patients received the same standard multi-modal therapy, including PT, OT, ST, maximum of 300 min per day individualized for each patient	placebo	Modified Ashworth Scale (MAS)	MAS +
Tao. 2015 [20]	23	<6 weeks	Botox®: 200 U	EMG	Comprehensive rehabilitation combined neurodevelopmental technique and motor relearning program encompassing PT (45 minutes per day) OT (30 minutes per day) and gait training.	placebo	Modified Ashworth Scale (MAS) Fugl-Meyer score (FMS) Modified Barthel Index (MBI) Walking speed (WS)	MAS + FMS + MBI + WS +
Jiang Li. 2017 [26]	104	NA	HengLi®: 200 U, 400 U.	ES	No rehabilitation program was recorded.	dose/ concentration ranging	Modified Ashworth Scale Gait velocity	400 U (100 U/ml) group showed better results.
Gracies. 2017 [23]	388	>6 months	Dysport®: 1000 U, 1500 U	ES	No standardized physiotherapy regimen was associated with this protocol.	placebo/ dose-ranging	Modified Ashworth Scale (MAS) Physician global assessment (PGA) Gait velocity (GV)	MAS + PGA – GV –
Wein. 2018 [21]	468	>3 months	Botox®: 300–400 U	EMG and/ or ES, ultrasound	No rehabilitation program was recorded.	placebo	Modified Ashworth Scale (MAS) Clinical Global Impression of Change (CGI) Goal Attainment Scale (GAS)	MAS + CGI + GAS +
Kerzoncuf. 2019 [22]	40	>12 months	Botox® < 300 U Mean:227 U	ES	Patients continued their rehabilitation programs which were not systematically recorded.	placebo	Modified Ashworth Scale (MAS) Sway area (SA) Gait velocity (GV) Functional Ambulation Classification (FAC) Functional Independence Measure (FIM)	MAS + SA + GV – FAC – FIM –

The table shows the characteristics of selected studies. The characteristics of studies included the first author’s name and published year, the number of participants, event duration (NA: Not available), preparations and doses (U: unit) of BoNT-A, the use of injection guidance techniques (EMG: Electromyography, ES: electrical stimulation), concomitant rehabilitation program (PT: Physical therapy, OT: Occupational therapy, ST: Speech and language therapy), outcome measures, and results: (+) indicates significant improvements in the BoNT-A group compared to the placebo group, (–) indicates nonsignificant improvements in the BoNT-A group compared to the placebo group.

**Table 2:** Dosage regimens of individual trials using Botox®.

First Author, Year	Total Dose	Gastrocnemius		Soleus	TP	FDL	FDB	FHL	EHL	Reconstituted
		Medial	Lateral							
Mancini.2005 *	167 U	50 U		50 U	50 U	50 U	50 U			100 u/2 mL
	322 U	100 U		75 U	100 U	75 U	75 U			
	540 U	200 U		100 U	200 U	100 U	100 U			
Kaji.2010	300 U	75 U	75 U	75 U	75 U					100 U/5 mL
Dunne.2012	200 U	50 U		80 U	70 U					20 U/mL
	300 U	75 U		125 U	100 U					
Pimentel.2014	100 U	50 U	50 U							100 U/2 mL
	300 U	100 U	100 U	100 U						
Fietzek.2014	230 U	60 U	30 U	70 U	70 U					100 U/2 mL
Tao.2015	200 U	50 U	50 U	50 U	50 U					NA
Wein.2018	300–400 U	75 U	75 U	75 U	75 U	≤100 U * into optional muscles				100 U/4 mL
Kerzoncuf.2019 *	227 U	50–100 U		50–160 U	50–100 U	50–100 U		25–50 U	25 U	NA

Abbreviations: U: unit, TP: tibialis posterior, FDL: flexor digitorum longus, FDB: flexor digitorum brevis, FHL: flexor hallucis longus, EHL: extensor hallucis longus. NA: Not available. *: individualized for each patient.

**Table 3:** Dosage regimens of individual trials using Dysport®.

First Author, Year	Total Dose	Gastrocnemius		Soleus	TP	FDL	FDB	FHL	EHL	Proximal Muscles	Reconstituted
		Medial	Lateral								
Burbaud.1996 *	1000 U	500–1000 U		200–400 U	200–350 U	150–300 U					1000 U/5 mL
Pittock.2003	500U	1.5/4	1/4	1.5/4							4 mL
	1000 U										
	1500 U										
Gracies.2017 *	1000 U	1.5/7.5		2.5/7.5	The remainder of the dose was injected into other muscles selected by the investigator.						7.5 mL
	1500 U										

Abbreviations: U: unit; TP: tibialis posterior; FDL: flexor digitorum longus; FDB: flexor digitorum brevis; FHL: flexor hallucis longus; EHL: extensor hallucis longus; *: individualized for each patient; Proximal muscles: Rectus femoris, Hamstrings, Gluteus maximus, Adductor magnus, Gracilis.

## Data Availability

Data is contained within the article or Supplementary Material.

## Supplementary Materials

The following are available online at https://www.mdpi.com/article/10.3390/toxins13060428/s1, Figure S1—Funnel plot of muscle tone assessment at all time points, Figure S2—Forest plot of muscle tone assessments at week four after treatment (subgroup analysis), Table S1—The quality of studies basing on the PEDro criteria.

## Appendix A. Search Terms

### Appendix A.1. Pubmed

“Botulinum Toxins, Type A” [Mesh] AND (“lower limb spasticity” OR “lower extremity spasticity” OR “spastic lower limb” OR “lower limb muscles” OR “spastic equinus” OR “Spastic Equinovarus” OR “spastic foot”).

### Appendix A.2. Cochrane

#1 Mesh descriptor: [Botulinum Toxins, Type A] explode all trees.

#2 (“lower limb spasticity” OR “spasticity of the lower limb” OR “lower extremity spasticity” OR “spastic lower limb” OR “lower limb muscles” OR “spastic equinus” OR “spastic Equinovarus” OR “spastic foot”):ti,ab,kw.

#1 AND #2.

### Appendix A.3. Embase

(‘botulinum neurotoxin type a’:ab,ti OR ‘clostridium botulinum a toxin’:ab,ti OR ‘botulinum toxin a’:ab,ti) AND (‘lower limb spasticity’:ab,ti OR ‘spasticity of the lower limb’:ab,ti OR ‘lower extremity spasticity’:ab,ti OR ‘spastic lower limb’:ab,ti OR ‘lower limb muscles’:ab,ti OR ‘spastic equinus’:ab,ti OR ‘spastic equinovarus’:ab,ti OR ‘spastic foot’:ab,ti).

### Appendix A.4. Web of Science

((botulinum toxin A OR Botulinum Neurotoxin Type A OR Clostridium botulinum A Toxin OR OnabotulinumtoxinA OR Neurotoxin A) AND (lower limb spasticity OR spasticity of the lower limb OR lower extremity spasticity OR spastic lower limb OR lower limb muscles OR spastic equinus OR spastic Equinovarus OR spastic foot)).

### Appendix A.5. CINAHL

botulinum toxin type A AND “lower limb spasticity” OR “spasticity of the lower limb” OR “lower extremity spasticity” OR “spastic lower limb” OR “lower limb muscles” OR “spastic equinus” OR “spastic Equinovarus” OR “spastic foot”.
