# Supplementary material for: Efficacy and Optimal Dose of Botulinum Toxin A in Post-Stroke Lower Extremity Spasticity: A Systematic Review and Meta-Analysis
Source: Toxins (Basel). 2021 Jun 18;13(6):428. doi: 10.3390/toxins13060428 (PMC8234518; doi:10.3390/toxins13060428)
Supplement: Supplementary file 1 [file toxins-13-00428-s001.zip › toxins-1256249-supplementary.pdf]

Supplementary Materials: Biofilm-Forming Methicillin-Resistant *Staphylococcus aureus* Survive in Kupffer Cells and Exhibit High Virulence in Mice

Thanh-Nhan Doan, Mei-Ying Kuo and Li-Wei Chou

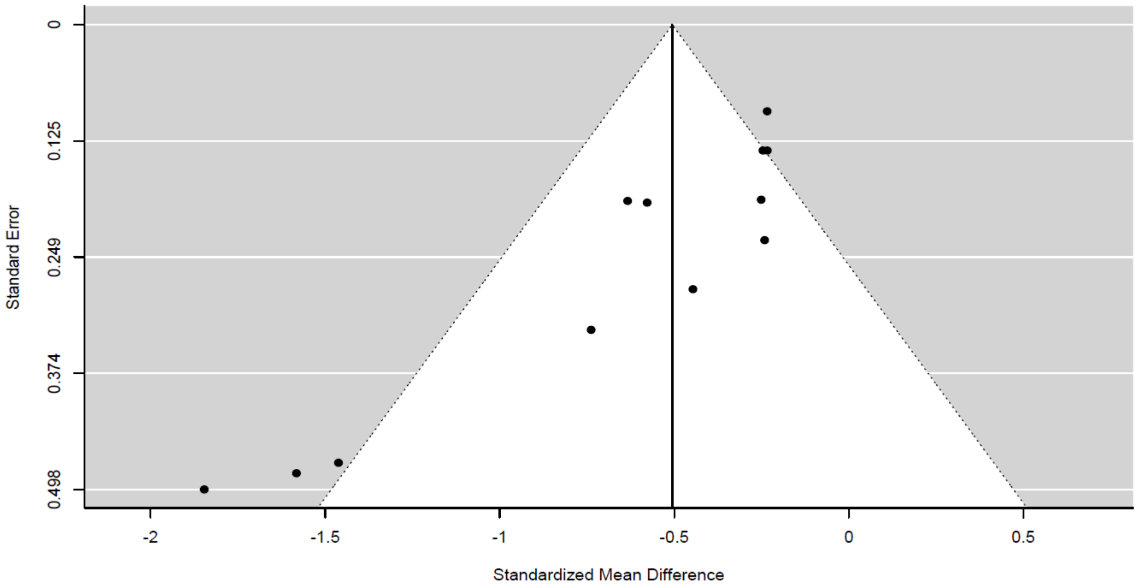

Figure S1. Funnel plot of muscle tone assessment at all time points.

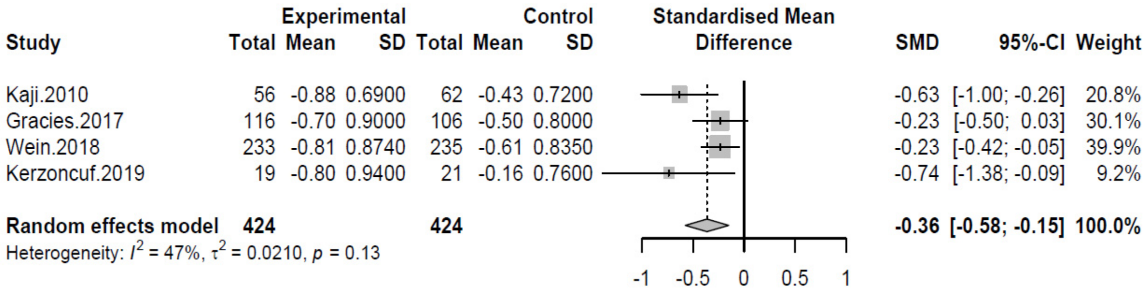

Figure S2. Forest plot of muscle tone assessments at week four after treatment (subgroup analysis).

Table S1. The quality of studies basing on the PEDro criteria.

Study	Specified eligibility criteria	Random allocation	Concealment of allocation	Comparable at baseline	Blinding of subjects	Blinding of therapist	Blinding of assessors	key outcome data for at least 85% of the subjects	Analysis by intention to treat	Between group analysis	Point estimate and variability	Total PEDro Score
Burbaud.1996	1	1	1	1	1	1	1	1	1	1	1	10
Pittock.2003	1	1	1	1	1	1	1	1	0	1	1	9
Mancini.2005	1	1	1	1	1	1	1	1	1	1	1	10
Kaji.2010	1	1	1	1	1	1	1	1	0	1	1	9
Dunne.2012	1	1	1	1	1	1	1	1	0	1	1	9
Pimentel.2014	1	1	1	1	1	1	1	1	0	1	1	9
Fietzek.2014	1	1	1	1	1	1	1	1	0	1	1	9
Tao.2015	1	1	1	1	1	1	1	1	1	1	1	10
Jiang Li. 2017	1	1	1	1	1	1	1	1	1	1	1	10
Gracies.2017	1	1	1	1	1	1	1	1	0	1	1	9
Wein.2018	1	1	1	1	1	1	1	1	0	1	1	9
Kerzoncuf. 2019	1	1	1	1	1	1	1	1	0	1	1	9
